# Posttraumatic Stress Symptoms after Exposure to Two Fire Disasters: Comparative Study

**DOI:** 10.1371/journal.pone.0041532

**Published:** 2012-07-24

**Authors:** Nancy E. Van Loey, Rens van de Schoot, Albertus W. Faber

**Affiliations:** 1 Department of Psychosocial and Behavioural Research, Association of Dutch Burns Centres, Beverwijk, The Netherlands; 2 Department of Methods and Statistics, Utrecht University, Utrecht, The Netherlands; 3 Department of Clinical Psychology, Martini Hospital, Groningen, The Netherlands; Institute of Psychiatry at the Federal University of Rio de Janeiro, Brazil

## Abstract

This study investigated traumatic stress symptoms in severely burned survivors of two fire disasters and two comparison groups of patients with “non-disaster” burn injuries, as well as risk factors associated with acute and chronic stress symptoms. Patients were admitted to one out of eight burn centers in the Netherlands or Belgium. The Impact of Event Scale (IES) was administered to 61 and 33 survivors respectively of two fire disasters and 54 and 57 patients with “non-disaster” burn etiologies at 2 weeks, 3, 6, 12 and 24 months after the event. We used latent growth modeling (LGM) analyses to investigate the stress trajectories and predictors in the two disaster and two comparison groups. The results showed that initial traumatic stress reactions in disaster survivors with severe burns are more intense and prolonged during several months relative to survivors of “non-disaster” burn injuries. Excluding the industrial fire group, all participants’ symptoms on average decreased over the two year period. Burn severity, peritraumatic anxiety and dissociation predicted the long-term negative outcomes only in the industrial fire group. In conclusion, fire disaster survivors appear to experience higher levels of traumatic stress symptoms on the short term, but the long-term outcome appears dependent on factors different from the first response. Likely, the younger age, and several beneficial post-disaster factors such as psychosocial aftercare and social support, along with swift judicial procedures, contributed to the positive outcome in one disaster cohort.

## Introduction

Throughout history, catastrophic fires have been an unfortunate occurrence in every society [1]. The devastating psychological impact of a massive fire disaster on persons’ psychological health was documented for the first time in survivors of the Cocoanut Grove disaster [2]. Although seven decades later a uniform definition of a disaster has yet to be accepted in the trauma literature, it is suggested that the large scale and a significant outcome in terms of mental and/or physical consequences, are two essential characteristics of a disaster [3].

Despite the number of fire disasters the subject of their potential psychological impact has received little attention. The scarce studies available, all being cross-sectional in nature, have investigated Posttraumatic Stress Disorder (PTSD), a central form of psychopathology associated with disasters [3]. Studies in this area have reported a prevalence rate of approximately 25% PTSD in burn survivors from hotel and discotheque fires respectively, 7 to 18 months after the disasters [4], [5]. Prospective longitudinal studies investigating posttraumatic stress symptoms in survivors with burn injuries sustained in fire disasters are currently not available.

It is generally assumed that posttraumatic stress symptoms gradually decrease after disasters, on average, as time passes and survivors are able to focus on normal activities of living. However, the course of PTSD after a disaster is unclear in empirical studies because of relatively short follow-ups (less than 1 year), which do not allow for the examination of long-term outcomes and delayed-onset PTSD [3]. Longitudinal studies that span a longer time period are necessary to study recovery patterns. Such studies may be especially necessary in major fire disasters because severe burn injuries typically cause long-term physical and functional limitations [6], and psychological and psychiatric problems that tend to persist for years [7], [8]. The complications associated with severe burn injuries pose additional threats to patient well-being, and may hamper comparisons with other disaster populations. Further, little is known regarding risk factors for chronic posttraumatic stress symptoms especially following fire disasters. In spite of evidence that pre-existing psychological problems are highly related to post-disaster adjustment [9], [10], a general trauma meta-analysis [11] showed that the variables present during and after traumas (e.g. trauma severity, additional life stress) have stronger effects. A possible indicator of subjective trauma severity might be peritraumatic dissociation [12], i.e. dissociative reactions experienced during or immediately after a trauma [13], [14]. Although still under debate, it is assumed that peritraumatic dissociation leads to a failure to integrate the trauma related information and prevents the realization that the trauma belongs to the past [13]. Nevertheless, more prospective studies are needed to clarify the relationship with PTSD.

Up till now, it has been unclear if disasters are more detrimental than individual traumatic events in terms of intensity and duration of posttraumatic stress symptoms. Based on the dose-response model, which holds that posttraumatic stress worsens with the severity of the stressor [15] it might be argued that experiencing a disaster constitutes a higher risk of developing PTSD than sustaining a injury through most individual accidents. Supporting the validity of this model, the highest prevalence of PTSD has been reported among samples of survivors with close proximity to disasters and higher exposure (e.g., duration) to traumatic events [3], [16]. However, this model is also controversial because the long-term outcomes of PTSD are often inconsistent with the initial event’s magnitude, and other factors seem to contribute more heavily to the outcome [11].

To our knowledge, no study has assessed traumatic stress symptoms in the immediate aftermath of a fire disaster, and examined their relationship to longitudinally measured and long term follow-up. We studied the trajectory of traumatic stress symptoms, and the early predictors of traumatic stress symptoms, in two samples of patients that survived two separate human-made fire disasters that resulted in mass burn casualties. Moreover, we compared their traumatic stress symptoms to two groups of patients who received burns injuries in individual (non-disaster) accidents, and were comparable regarding gender, age, and burn severity. We used latent growth modeling in a longitudinal design involving 4 groups of patients. This sophisticated data analytic approach has been particularly useful in longitudinal research because different trajectories can be identified [17]. We hypothesized that the survivors of the fire disasters would not only have higher traumatic stress levels compared to those of individual accidents, but would have them over a longer period of time.

## Methods

### Ethics Statement

The study was conducted in accordance to the ethical principles of the Helsinki declaration and ethical approval was obtained from the Ethics committee of the University of Ghent, Belgium.

### Description of Disasters

The first disaster, a café fire, happened on January 1, 2001. The event took place in Volendam, a village near Amsterdam. Hundreds of adolescents were celebrating New Year’s Eve in a café on the third floor of a small house. Shortly after midnight, the partygoers lit sparklers which accidentally set fire to Christmas decorations hanging from the ceiling. The fire resulted in 245 survivors, of which 215 had severe burns. Seventy-eight patients were taken to burn centers across the country and to Belgium and Germany. Four people died at the scene, and ten more lost their lives in the following days, weeks and months [18].

The second disaster, an industrial fire, took place on July 30, 2004, in Ghislenghien, 40 km southeast of Brussels. Road construction caused a gas leak. As a consequence, a massive natural gas pipeline exploded and ravaged the surrounding environment. It melted or burned everything within a 400-metre radius and left a crater, 8–10 m in diameter and 3–4 m deep. Many workers in the industrial park received burn injuries, as did passers-by on the nearest highway. The explosion resulted in 132 wounded, of which 65 were transported to burn centers in Belgium and France. A total of 24 people died at this site [19].

### Participants

#### The fire-disaster exposed groups

We included 61 patients involved in the café fire (25% of the total wounded). Of these patients, 46 were treated in burn centers and 15 were treated in general or academic hospitals. We collected data from this latter group at the outpatient clinic where they received medical follow-ups from one of the burn centers. These 61 patients were comparable with regard to age and gender ratio to the 245 total wounded; however, the study participants had more severe injuries in terms of mean total body surface area burned (TBSA) (total  = 12% versus study sample  = 25%) and number of patients with inhalation injury (total  = 36% versus study sample  = 67%) [18]. The 33 patients involved in the industrial fire were all treated in burn centers (25% of the total wounded). We were not able to find a published report on the injury and demographic characteristics of the total wounded in the industrial fire group. It is probable, however, that this disaster group also included patients with more severe burn injuries.

#### The comparison groups

We selected two comparison patient groups drawn from the same studies that were comparable with the disaster cohorts regarding TBSA burned, age, and gender. In accordance with the characteristics of the respective disaster groups, we used the following inclusion criteria. Comparison Group 1 consisted of patients younger than 26 years old with a TBSA of more than 4%. Comparison Group 2 consisted of patients between 27 and 52 years old with a TBSA of more than 10%. The male/female ratio was comparable between disaster and comparison groups. This selection resulted in two separate comparison groups that consisted of 54 and 57 patients who were burned in individual accidents. The attrition rate did not differ considerably across the disaster groups and their respective comparison groups. Table 1 shows the descriptive statistics for all four groups.

**Table 1:** Group descriptive statistics.

	Café fire group(N = 61)	Comparison café fire(N = 54)	Industrial fire group(N = 33)	Comparison industrial fire(N = 57)
**Demographics**				
Male	**39 (63.9%)**	38 (70.4%)	**29 (87.9%)**	48 (84.2%)
Age (M/SD)	**17.6 (2.0)**	19.9 (3.1)*	**40.2 (10.4)**	38.0 (8.7)
**Injury characteristics**				
TBSA (M/SD)	**25.4 (15.5)**	21.0 (13.0)	**28.2 (11.4)**	28.2 (15.0)
LOS (M/SD)	**49.8 (41.9)**	31.4 (26.1)*	**55.6 (39.8)**	44.0 (34.2)
Inhalation injury	**44 (72.1%)**	8 (14.8%)*	**8 (24.2%)**	19 (33.3%)
**Self-report measures**				
ADS anxiety (M/SD)	**57.9 (21.1)**	47.4 (24.2)*	**63.1 (24.5)**	47.9 (27.5)*
ADS dissociation (M/SD)	**48.3 (26.9)**	30.3 (28.9)*	**57.1 (28.2)**	31.4 (30.7)*
Idea not surviving	**49 (80.3%)**	17 (31.5%)*	**28 (84.8%)**	22 (38.6%)*
No return work/school 3 mth	**30 (54.5%)**	24 (44.4%)	**16 (48.5%)**	38 (66.7%)
No return work/school 12 mth	**10 (16.5%)**	9 (16.7%)	**14 (42.4%)**	14 (24.6%)*
**Response rates**				
Response rate 12 months	**46 (75.4%)**	46 (82.1%)	**27 (81.8%)**	51 (89.5%)
Response rate 24 months	**30 (49.2%)**	31 (44.6%)	**17 (51.5%)**	30 (49.2%)

* Statistically significant differences between disaster group and its comparison group (p<.01); TBSA: Total Body Surface Area burned, LOS: Length of stay in hospital.

### Procedures

The present study is part of a larger investigation. Between 1997 and 2005, we conducted prospective cohort studies in Dutch and Belgian burn centers to examine posttraumatic stress symptoms in patients with burn injuries. The full details of these studies have been published elsewhere [20], [21]. The aforementioned fire disasters took place during the above study periods which allowed for a comparison of the survivors of these disasters with those sustaining burns through individual accidents.

A local researcher invited patients to participate in our study between 3 and 7 days after the burn event or as soon as they gained sufficient cognitive alertness after a period of artificial ventilation or delirium. All patients, and parents of patients who were younger than 18 years of age, provided written informed consent. Once patients left the burn center, the local researcher continued to pursue data collection with participants by mail. Follow-up questionnaires were sent to their home address and included a pre-paid return envelope and a personal letter.

### Measures

#### Posttraumatic stress symptoms

The Impact of Event Scale (IES) [22] assessed posttraumatic stress symptoms on five occasions: 2 weeks as well as 3, 6, 12, and 24 months post-burn. The IES is a validated 15-item self-report scale that assesses two PTSD symptom-clusters: intrusive and avoidant symptoms [23] and is widely used across the world to measure traumatic stress symptoms. The IES discriminates between people with PTSD and those without [24]. This study used the Dutch version of the IES [25]. Answers were scored on a scale ranging from 0 to 100. We used a cut-off score of 33 to indicate severe stress responses [20].

#### Peritraumatic anxiety and dissociation

The Anxiety and Dissociation Scale (ADS) measures participants’ subjective responses during or immediately following the traumatic event. The measure was completed during the first post-burn week. The seven questions in this measure address the intensity of participant dismay, panic, yelling, trembling with fear, depersonalisation, derealisation and time distortion, which are rated on a visual analogue scale from 0 (*not at all*) to 100 (*the worst imaginable way*). The scale includes two subscales: anxiety and dissociation. The ADS’s psychometric properties are satisfactory [20].

#### Demographic data and injury severity

Additional data collected included participants’ age and gender, and injury characteristics such as TBSA (i.e., the estimated percentage of partial and full thickness burns). We also examined whether participants had returned to work or school at 3 and 12 months.

### Statistical analysis

First, we conducted a comparison between the descriptive characteristics of the four groups (two disaster and two comparison groups) using T-tests and χ^2^-tests including the mean longitudinal stress scores. Second, we examined latent growth trajectories in the total sample (thereby ignoring the four groups) to identify classes of individuals with different response trajectories during a 2-year period. Because each of the four groups was too small to investigate the response classes separately, we identified participants’ trajectory of traumatic stress symptoms in the four groups using cut-off scores [20] in accordance with the patterns identified in the sophisticated analytic approach. Last, we conducted a four-group latent growth modeling (LGM) [26] to analyze the changes in posttraumatic stress scores over time, and to identify predictors in the separate groups. In this approach LGM enabled us to examine individual growth trajectories for each of the four study groups. Specifically, within each group individuals began with a different starting point (i.e., a random intercept model) and had a different growth rate (i.e., a random slope model) allowing us to explain variance within the groups. This way for each of the four groups different predictors explain variance around the intercept or slope. The R-squared statistic provides the proportion of variance in posttraumatic stress symptoms that is explained by the latent growth factors. Missing data were estimated using the full information maximum likelihood method. Furthermore, we applied a robust maximum likelihood estimator because some of the variables were not normally distributed (e.g., IES, anxiety and dissociation). We used Mplus 6.11 [27], a statistical program for the analyses of latent variable models of which LGM is one of the model features.

## Results

### Descriptive Statistics, Prevalence and Course of Posttraumatic Stress Symptoms

**Figure 1:**
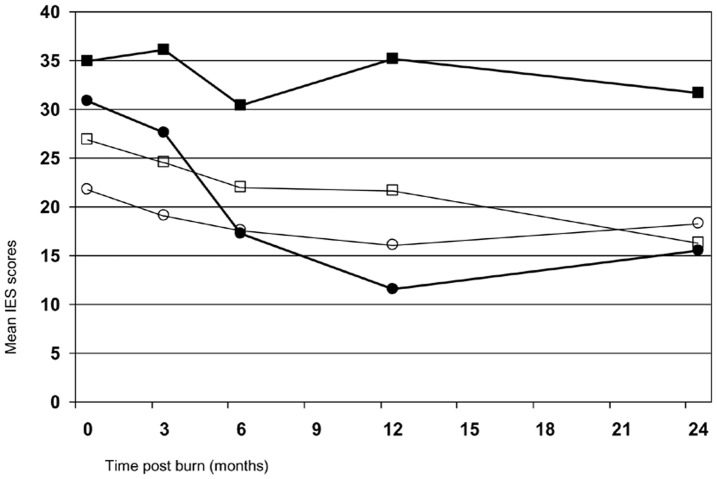
Observed mean IES scores in four groups during the two year follow-up. The café fire group is depicted by the black circles, its comparison group by the white circles. The industrial fire group is depicted by the black rectangles, its comparison group by the white rectangles.

Table 1 shows that both disaster groups reported significantly higher peritraumatic anxiety and dissociation scores, and they were more likely to believe that they would not survive the accident, which illustrates how the disasters might have a higher psychological impact compared to regular burn accidents. There were no differences with regards to return to work or school at 3 months. However, at 12 months, 42% of the industrial fire did not return to work which is higher than the other groups. Figure 1 presents the mean IES scores for the four groups. T-tests indicate that the disaster groups had higher initial traumatic stress scores compared to their respective comparison group at the first two measurements. At 2-weeks post-burn, the café fire group had a mean IES score of 30.8 (SD  = 17.8), whereas its comparison group had a score of 22.1 (SD  = 16.0; t  = 2.8, df  = 111, p  = .007). At 3-months post-burn, the scores were 27.5 (SD  = 19.0) v. 18.8 (SD  = 14.2; t  = 2.6, df  = 105, p  = 0.009). Compared to its comparison group, the industrial fire group reported higher IES scores at 2 weeks (M  = 34.9 (SD  = 21.9) v. M  = 26.9 (SD  = 18.3); t  = 1.9, df  = 88, p  = .068) and 3 months (M  = 36.1 (SD  = 22.6) v. M  = 24.5 (SD  = 21.5); t  = 2.6, df  = 80, p  = .026). At 6-months post-burn, both fire-disaster groups’ stress scores did not differ statistically significantly from their respective comparison group (t-tests not reported). However, the mean scores of the industrial fire group increased again after 6 months and remained significantly higher than their comparison group at 2 years post-burn.

### Latent Growth Trajectories Investigating Response Classes

**Figure 2:**
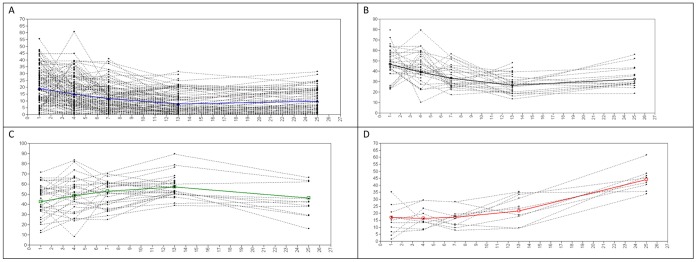
Estimated means and observed individual values representing four classes of response patterns. 2a. Low stress trajectory (resilience); 2b. Acute stress trajectory, 2c. Chronic stress trajectory, 2d. Delayed onset trajectory.

In Figure 2 we present the four latent classes that could be identified in this data set (n  = 207) showing the response patterns present in the total sample. As can be seen in Figure 2a, the majority of individuals (n  = 130, proportion  = .628) had relatively low scores. Figure 2b and 2c show the trajectory of individuals with acute stress scores that decreased over time (n  = 36, proportion  = 0.174) and rather high and chronic stress scores (n  = 31, proportion  = 0.150) respectively. Finally, in Figure 2d a small group of 10 individuals (proportion  = 0.048) showed increased scores, indicating a delayed onset of posttraumatic stress scores.

### Descriptive Analyses of Response Classes in the Two Disaster and Two Comparison Groups

**Figure 3:**
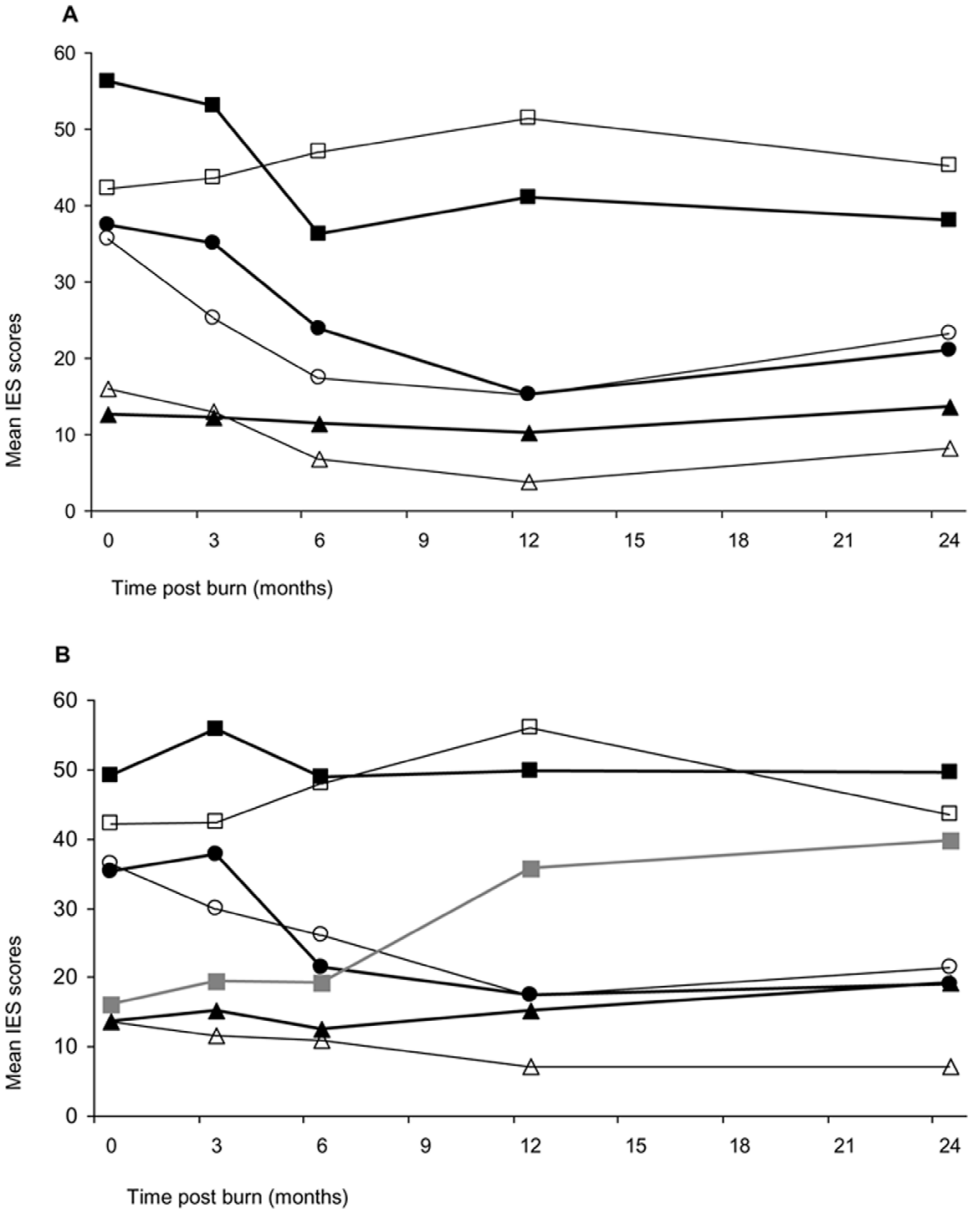
Trajectories of traumatic stress symptoms. (A) Represents trajectories in the café fire group and its comparison group; (B) Represents trajectories in the industrial fire group and its comparison group. Chronic stress trajectory is depicted by black and white rectangles, acute stress trajectory is depicted by circles, resilient trajectory is depicted by triangles, delayed onset trajectory is depicted by grey line. Thick lines represent stress scores in fire disaster survivors, thin lines represent stress scores in regular burn survivors.

Because the samples within each group (two disaster and two comparison groups) are too small to investigate these response patterns, we described these trajectories using a cut-off score [20]. The trajectory “acute stress” includes patients who have high initial stress scores but who score below the cut-off point after 6 months. The trajectory “chronic stress” includes patients with scores above the cut-off for the entire study period, whereas patients with the trajectory “low stress scores” never surpassed the cut-off point. Finally, the “delayed onset” trajectory includes patients who had initial scores under the cut-off point but surpassed it after 6 months. Figure 3a and 3b shows the trajectories of the two disaster groups and their respective comparison groups. These figures demonstrate that individuals in both disaster groups who developed chronic stress reactions, had high initial stress scores. Furthermore, both disaster groups showed delays in recovery as part of the acute stress trajectory during the first three months, whereas both comparison groups showed an immediate decrease in stress. Note that – by using the cutoff score – the delayed onset trajectory occurred only in the industrial fire group.

Both comparison groups showed similar percentages of patients with acute stress trajectories (n  = 10; 19 and 17% respectively), although the younger comparison group comprised a larger proportion of people (n  = 33 (61%) versus n  = 28 (49%)) who did not report clinically relevant posttraumatic stress symptoms. The disaster groups were clearly differentiated from each other with regard to the proportion of people within the different trajectories. Twenty-one patients (40%) in the café fire group had high initial stress scores that decreased below the cut-off score whereas only three patients (10%) in the industrial fire group showed a similar pattern. In addition, three people (10%) in the industrial fire group showed a delayed onset of traumatic stress symptoms, which resulted in 16 patients (55%) who suffered from chronic stress reactions 1 year after the disaster; conversely, only four patients (8%) suffered from severe posttraumatic stress symptoms 1 year later in the café fire group.

### Four-group Latent Growth Modeling

Table 2 presents the mean intercepts, slopes, and standardized estimates for the multi-group LGM analysis of the traumatic stress scores. The first model evaluated the 24-month longitudinal traumatic stress scores for the four groups. A basic quadratic growth model of the traumatic stress scores produced an adequate fit (CFI  = 0.96, TLI  = 0.96, RMSEA  = 0.09). All groups except the industrial fire group, demonstrated a significant decrease in stress scores. Additionally, the café fire group and its comparison group showed a small but significant quadratic effect, which indicates that stress scores increased again over time but at a much smaller rate compared to the initial decrease. Next, we added gender, age, TBSA, peritraumatic anxiety, and dissociation as predictors in the LGM model to explain the individual differences in initial mean and change over time. However, this model failed to produce a sufficient fit.

**Table 2:** Mean estimated IES scores in two disaster groups and two comparison groups over time and predictors (only 12 months study period).

	Café fire group(N = 61)	Comparison café fire(N = 54)	Industrial fire group(N = 33)	Comparison industrial fire(N = 57)
**24 months**	**Estimate (s.e.)**	Estimate (s.e.)	**Estimate (s.e.)**	Estimate (s.e.)
Intercept (mean)	34.035 (2.182)**	22.279 (2.173)**	35.817 (3.688)**	25.153 (2.582)**
Slope (mean)	−2.614 (0.244)**	**−**0.877 (0.310)**	**−0.317 (0.386)**	**−**0.207 (0.077)**
Quadratic term (mean)	0.079 (0.008)**	0.032 (0.012)**	**0.017(0.013)**	0.000 (0.000)
**12 months**				
Intercept (mean)	31.066 (2.073)**	20.837 (1.900)**	35.179 (3.854)**	26.083 (2.313)**
Slope (mean)	−1.384 (0.135)**	**−**0.354 (0.173)**	**−0.039 (0.248)**	**−**0.339 (0.186)*
Intercept on:				
TBSA	**−0.037 (0.153)**	0.148 (0.150)	−0.386 (0.159)**	**−**0.194 (0.123)
Gender	**−0.147 (0.144)**	**−**0.117 (0.148)	**0.136 (0.155)**	0.094 (0.467)
ADSanx	0.436 (0.144)**	0.124 (0.152)	0.638 (0.177 )**	0.586 (0.117)**
ADSdis	**0.224 (0.159)**	0.443 (0.142)**	**−0.135 (0.206)**	0.367 (0.138)**
Slope on:				
TBSA	**−0.054 (0.496)**	0.265 (0.327)	0.523 (0.213)**	0.143 (0.202)
Gender	**0.345 (0.547)**	**−**0.541 (0.325)*	**0.108 (0.195)**	0.428 (0.254)*
ADSanx	**−0.735 (0.458)**	0.206 (0.278)	−0.746 (0.255)**	**−**0.053 (0.210)
ADSdis	**−0.334 (0.586)**	**−**0.228 (0.298)	0.955 (0.273)**	**−**0.364 (0.286)
R Square				
Intercept	**0.36**	0.27	**0.51**	0.58
Slope	**-**	0.54	**0.58**	0.25

* one tailed tested; ** two tailed tested.

As such, we analyzed the LGM model again over a 12-month period. We began with the basic model showing that a linear growth model of stress scores produced an adequate fit (CFI  = 0.98, TLI  = 0.98, RMSEA  = 0.10). Table 2 shows that, in line with their 24-month outcomes, the stress scores of all participants except those in the industrial fire decreased. The quadratic term in the 12-month period was not significant nor did it fit the data, which demonstrates that a small increase in stress scores occurred between 12 and 24 months after the event.

We added the same predictors as before to the model. Because age was not significantly related to the traumatic stress scores for any of the groups, we removed this variable from further analyses; thus, the LGM model of traumatic stress scores during the first 12 months consisted of TBSA, gender, anxiety, and dissociation (CFI  = 0.95, TLI  = 0.91, RMSEA  = 0.11). Moreover, the model improved according to the trade-off between model fit and model complexity as measured by the Bayesian information criterion [28] (BIC change  = 106.769). The four predictors explained a relatively large proportion of the variance in the initial stress scores measured 2 weeks post-burn for all the groups. Our results indicate that peritraumatic anxiety, dissociation, or both partly predicted the initial stress scores in the four groups. Remarkably, anxiety symptoms but not dissociation significantly predicted the initial stress response in both disaster groups. Also, larger burn severity was not associated with higher acute stress scores. Furthermore, these independent variables were able to predict a significant proportion of the variance in change over time (i.e., slope) for the industrial fire cohort and the comparison groups, but not for the café fire group (See Table 2). In contrast to the other groups, burn severity and peritraumatic reactions predicted the change over time in the industrial fire group; anxiety symptoms predicted a higher decrease in stress symptoms whereas dissociation symptoms predicted a higher increase over time. Gender was statistically significantly related to the change over time in the two comparison groups, be it in opposite directions (one-tailed test). No predictor could explain the rate of change in the café fire group.

## Discussion

There are a number of unique features of the present study, including the use of disaster burn samples compared to individual etiologies of trauma, prospective early and longitudinal measure administration that avoids retrospective memory bias, and follow-up lasting as long as two years. The study yielded two important conclusions regarding acute and long-term traumatic stress symptoms of survivors of mass-casualty fire disasters. First, disaster survivors showed higher levels of peritraumatic anxiety and dissociation, and more severe symptoms of traumatic stress and over a longer period of time than did regular burn survivors. Whereas the traumatic stress symptoms in survivors of individual accidents largely decreased after 3 months, survivors of fire disasters still suffered from higher stress during this period. This finding supports a dose-response effect in the immediate aftermath of disasters and suggests that at least some of the acute stress symptoms that follow fire disasters result from the event itself. Second, the finding that the two groups of disaster survivors had different long-term outcomes, supports a large literature that challenges the dose-response effect on the longer term [11]. Despite the comparable high impact context and initial stress levels, a large proportion of the café fire group survivors had scores that returned to a level that was below the cut-off which is in sharp contrast to the majority of survivors in the industrial fire group which maintained a high level of distress during the entire study period. Apparently a dose-dependent model does not explain all of the psychological reaction after trauma and may underscore the role of individual variability [29].

Findings from latent growth modelling showed psychological recovery and resilience in many severely burned survivors, and only a minority showed evidence of chronic or delayed onset traumatic stress symptoms. The identification of the multiple response patterns using sophisticated analytic procedures replicated earlier findings in other populations [30]. However, on average, the industrial fire victims in the current study showed a stable pattern of stress symptoms over time in line with a French industrial fire study [31]. Interestingly, the café fire and its comparison group showed an overall decrease over time but also a small but significant increase in stress symptoms occurring after 12 months, indicated by the quadratic slope term. Because it is a small effect, its clinical relevance may be debatable. On the other hand, it may indicate that traumatic stress scores can increase again after a certain period of time.

The risk factors peritraumatic anxiety and/or dissociation partly predicted the baseline level of stress scores in all four groups, be it that in the disaster groups dissociation was not significantly associated with baseline stress scores. Burn severity did not seem to immediately affect the stress scores; in the industrial fire there was even a negative relationship with stress scores at baseline. Furthermore, the course of stress scores in the industrial fire group was predicted by TBSA, dissociation, and anxiety. Those with higher levels of dissociation and with more severe burns showed an increase in stress scores over time whereas higher anxiety symptoms predicted a higher decrease in symptoms over time. These findings are in line with a large number of studies that consistently showed a positive relationship between peritraumatic dissociation and posttraumatic stress symptoms [11], [14], [20], [31]. The reason why these associations were not found in the comparison groups may relate to a lack of statistical power due to lower prevalence rates of high peritraumatic anxiety and dissociation. This explanation does not hold for the café fire group that seems to be ‘the odd man out’. In both comparison groups, gender was a statistically significant predictor for the time course, be it that in the young comparison group males were more likely to maintain higher stress scores whereas in the older comparison group, female gender was a risk factor. The literature is quite consistent in its findings that female gender is a risk factor for posttraumatic stress [32]. The contradictory finding in the young comparison group may be an incidental finding due to the small sample size. Alternatively, it may indicate that the impact of gender on the vulnerability to develop posttraumatic stress symptoms increases with older age along with a higher exposure to traumatic events in general.

As the previous predictors fall short to explain the different outcomes, in particular the positive outcome in the café fire survivors, addressing the differences on group level between the café fire survivors and the industrial fire survivors may add to our understanding of these results. First, patients in the café fire group were all under 26 years old, in contrast to the middle-aged people in the industrial café fire group. The age-matched comparison group of the café fire, a burn study [33], and tsunami and earthquake studies [10], [34] all pointed to a greater likelihood of chronic traumatic stress symptoms in older people. These findings suggest a greater resilience in young people and may explain the better recovery from the high initial stress levels in the young café fire group. Second, all survivors of the café fire lived in the place where the disaster happened. The community empowerment and companionship between the victims may have increased social support. This may be especially relevant in burn survivors who are faced with lifelong scarring and associated stigmatization in social situations [35]. A third difference between the two disasters relates to the speed of the litigation process. The litigation process for the café fire was completed within the study period, whereas this process had not even made a start in the industrial fire. At the legal proceedings, responsibility for the event was attributed and the survivors of the café fire received financial compensation. Other studies have reported that lingering litigation proceedings [36] and financial problems [37] after an accident predicted the persistence of PTSD. Four, disparities in aftercare procedures might affect the outcomes. In line with recommendations of McFarlane [38], 2 days after the event the local authorities where the café fire took place created a long-term psychosocial aftercare. In support of the beneficial role of aftercare, the Belgian cabinet [39] acknowledged in an official press release that the needs for a regular psychosocial aftercare are unmet in the industrial fire survivors. Finally, 42% of the industrial fire group did not return to work at 12 months after the disasters. Although the reason why is unclear, it may have an economic impact and is therefore an additional negative life event beyond all other factors.

Summarizing, results of this study support the notion that empirical studies can not identify ‘a single dominant predictor’ of psychological outcome following disasters; rather, it is the combination of multiple factors that determines outcome [29]. Possibly, the combination of the younger age, high governmental and community support, and long-term aftercare facilities have significantly contributed to this favorable outcome in the café fire. Contrarily, the industrial fire group may have experienced a feeling of secondary victimization due to delayed litigation, lack of psychosocial aftercare, and economic difficulties that might have contributed to the maintenance of stress symptoms and might have elicited the delayed onset pattern in some individuals. To have experienced more subsequent negative life events has been associated with delayed onset PTSD in survivors of the World Trade Center Disaster [40].

This study suffers from limitations that should be mentioned. First, we used a self-report questionnaire to assess only two PTSD symptom clusters and we relied on a cut-off score to identify clinically significantly symptoms of stress. We tried to overcome the problems associated with cut-off scores by identifying long-term patterns to increase the reliability of our results. Despite the IES has good psychometric properties and is a useful and common measure to detect posttraumatic stress symptoms [24], [41] we can not determine the prevalence of a PTSD diagnosis. To give an impression, a recent study among burns survivors [42] reported a sensitivity and specificity of the IES of respectively 1 and 0.65, indicating that 65% of our sample scoring above the cut-off is likely to be diagnosed with PTSD (i.e., 51 (25% of total sample n  = 200) at baseline, 23 (14% of sample n  = 168) and 14 (14% of sample n  = 101) at 12 and 24 months respectively). Second, this study included only 25% of the casualties (specifically, those with more severe burns). Thus, our data may not generalize to all survivors. Third, the sample size within each group was small, in particular to run sophisticated analyses, which limits the number of predictors and affects the statistical power. Finally, the comparison groups were not case-matched. Such a matching procedure is preferable but would have further decreased the comparison groups’ sample size.

In conclusion, this study contributes to the literature by showing that the long-term traumatic stress outcome after two man-made fire disasters, despite comparable high initial stress responses, was quite different. This different outcome at group level may point to the importance of peri- and post-disaster factors in the recovery process. Although it remains speculative to what extent pre-trauma variables have played a role, this study suggests that post-disaster management plays a pivotal role in the adjustment process after disasters.
